# Argyrol in Eye-Work

**Published:** 1905-01-07

**Authors:** 


### ARGYROL IN EYE-WORK.

Mr. Sydney Stephenson[1] writes very enthusiastically of argyrol as a substitute for silver nitrate in eye-work. As is well known there are certain disadvantages attaching to the use of siver nitrate in inflammatory conditions of the eye, not the least of which is the pain which may be caused. Argyrol, Mr. Stephenson has found, is free from these drawbacks. Although it contains no less than 30 per cent. of metallic silver he has never known it cause the least pain, irritation, or inflammatory reaction. He has used the drug in all kinds of conjunctivitis, in blepharitis, in phlyctenular affections of the cornea and conjunctiva, and in affections of the lachrymal passages. Usually he has employed a 15 per cent. solution, and only in cases of gonococcal ophthalmia has he used a stronger solution, namely, one of 25 per cent. The weaker solution has been dropped into the eye three to eight times a day according to the kind and severity of the inflammation. The stronger solution has been painted over the exposed conjunctiva once or twice in the 24 hours. An argyrol ointment has been used also, chiefly in phlyctenular affections of the eye.

Not only does argyrol appear to act as a direct sedative to the inflamed conjunctiva, judging by the immediate relief produced; but no single patient, Mr. Stephenson states, has ever complained to him of any pain attending its use. Judging from the effect of argyrol on cases of acute contagious ophthalmia, its powers of penetration must be great. The symptoms of a sharp attack of acute conjunctivitis, due to the Koch-Weeks' bacillus, may be subdued in the course of two or three days without the least discomfort to the patient. It is equally effective in the treatment of corneal ulcers and blepharitis.

With the use of argyrol there is practically no fear of causing argyrosis, although in one case after the application of the drug from four to five times daily over a period of 144 days a very faint staining of the palpebral conjunctiva was noted. Although largin and protargol, which are somewhat similar preparations of silver, have many of the advantages of argyrol, yet they are both liable to cause staining of the tissues, and hence, if they are used, considerable caution must be exercised. On the whole, therefore, it appears best to make use of argyrol where a choice can be made.

[1] Med. Press, Dec. 21, 1904.

